# nPIV velocity measurement of nanofluids in the near-wall region of a microchannel

**DOI:** 10.1186/1556-276X-7-284

**Published:** 2012-05-31

**Authors:** Kanjirakat Anoop, Reza Sadr

**Affiliations:** 1 Micro Scale Thermo Fluids (MSTF) Laboratory, Mechanical Engineering Program, Texas A&M University at Qatar, P.O. Box 23874, Education City, Doha, Qatar

**Keywords:** nanofluids, nPIV, Newtonian fluid, viscosity, TIRF

## Abstract

Colloidal suspensions of nano-sized particles in a base fluid, nanofluids, have recently gained popularity as cooling fluids mainly due to their enhanced heat transfer capabilities. However, there is controversy in the literature on the reported properties of nanofluids and their applicability, especially since there is no fundamental understanding that explains these enhancements. A better understanding of these fluids and how they interact with a solid boundary may be achieved by a detailed near-wall fluid flow study at nanoscale. This work presents for the first time the near-wall velocity measurements for nanofluids using nanoparticle image velocimetry. This novel technique uses evanescent illumination in the solid–fluid interface to measure near-wall velocity field with an out-of-plane resolution on the order of O(100 nm). Nanofluids of different concentrations were prepared by dispersing silicon dioxide particles (10 to 20 nm) in water as the base fluid. Initially, viscosity measurements were conducted for the prepared nanofluids. The near-wall velocity data were then measured and compared with that of the base fluid at the same flow condition. It was observed that even though nanofluid viscosity had increased with particle loading, the near-wall velocity values were similar to that of the base fluid for a given flow rate. Together, these measurements vindicate the homogenous and Newtonian characteristics of the nanofluids in the near-wall region. Despite the low particle concentrations investigated, the present work also discusses the complexity involved in utilizing the methodology and possible errors arising during experimentation so as to implement this measurement tool more effectively in the future.

## Background

Thermal cooling is one of today’s most challenging technological problems, and there has been a constant effort by the scientific community to improve the heat transfer capabilities of cooling fluids. The notion that thermal conductivity of suspensions increases with the total surface area of the particles in the suspension triggered the concept of using nanosized particles in suspensions. This introduced a new class of engineered fluids called nanofluids: colloidal suspensions of nano-sized particles (approximately 5 to 100 nm) in a base fluid [1]. The prevalent popularity of nanofluids in the heat transfer community has been due to its reported anomalous thermal conductivity enhancement. Following the initial development of nanofluids, several experimental reports have shown the great potential of these fluids to be used for heat transfer applications [2-9]. However, there have been several recent reports questioning the heat transfer enhancement of nanofluids [10-16]. These reports assert that nanofluids behave as a homogeneous mixture and that their thermal properties can be successfully predicted by classical effective medium theories [13].

Several theoretical models and explanations have been proposed to explain the anomalous heat transfer characteristics of nanofluids. Brownian motion of nanoparticles in combination with aggregation and diffusion theories have been claimed to be the major justifications for the observed anomaly in thermal conductivity [17]. Flattening of velocity profile, shear thinning, and thermophoretic forces in the near-wall region have been asserted to be the probable causes for enhanced convective heat transfer characteristics of nanofluids [9,18,19]. However, it may be noted that most of the theoretically proposed models have not been directly validated by experimental results. Gaining an understanding of the near-wall flow field can help in better comprehending the phenomenon and assists in improving the theoretical models used in predicting the enhanced heat transfer characteristic of nanofluids. Walsh et al. [20] used microparticle image velocimetry (μPIV) [21] to obtain the velocity profile of nanofluids flowing inside a microchannel, *d* = 1,085 μm. However, the spatial, out-of-plane resolution of their work was from 4 to 30 μm and could not capture the flow velocities in the region very close to the wall. To the best of the authors’ knowledge, there have been no other experimental studies that have investigated the near-wall flow fields in nanofluids.

The evanescent wave-based PIV technique, also known as nanoparticle image velocimetry (nPIV), is an extension of nPIV which can substantially improve the out-of-plane resolution of the measurements close to the wall [22]. Evanescent wave-based techniques, namely total internal reflection microscopy and total internal reflection fluorescence (TIRF), have been widely used in biological and surface science fields in the past for near-wall visualization [23]. In nPIV, evanescent wave illumination generated by the total internal reflection (TIR) of a laser beam at the fluid–solid interface is used to illuminate particle tracers in the flow field with a spatial resolution on the order of O(100 nm). This technique has been successfully implemented in the past in fluid velocimetry for studying electro-osmotic flows through microchannels [24,25], near-wall Brownian diffusion [26], and the effect of the near-wall forces [27].

Present work demonstrates applicability of nPIV to investigate flow behaviors very near to the wall while using nanofluids. Near-wall measurements are reported for the first time for silicon dioxide (SiO_2_)-water nanofluid flow inside a microchannel at varying particle concentrations and flow rates with an out-of-plane spatial resolution of less than 300 nm. The results are then compared with those obtained for the base fluid. These results, along with the rheological characterization of the bulk nanofluids, are used to investigate occurrence of any non-homogeneity in the flow characteristics of the nanofluids in the near-wall region.

### Theory

Similar to PIV, both μPIV and nPIV ‘track’ naturally buoyant fluorescent tracers to measure fluid velocity with the assumption that they follow fluid flow faithfully. As mentioned earlier, in nPIV, the evanescent wave generated at the glass water interface is used to illuminate particles only in the near-wall region. A brief introduction into the working principle behind nPIV follows.

When a light beam travels through a medium with a refractive index *n*_1_ into another transparent medium with a lower refractive index of *n*_2_ at an angle exceeding the critical angle *θ*_c_ = sin^−1^(*n*_2_/*n*_1_), it is totally reflected at the interface. However, the electromagnetic field penetrates into the lower refractive index region and propagates for a small distance parallel to the interface creating what is called an evanescent wave. This evanescent wave is capable of exciting fluorescent particles in this region, while the large numbers of particles farther away in the bulk liquid remain unexcited. One distinct characteristic of the evanescence wave is its nonuniform intensity in the direction normal to the interface, where the intensity, *I*, decays exponentially with distance, *z*, normal to the wall as follows:

(1) $$I=I_{0}\exp\left(-z/z_{p}\right)\text{.}$$

**Figure 1:**
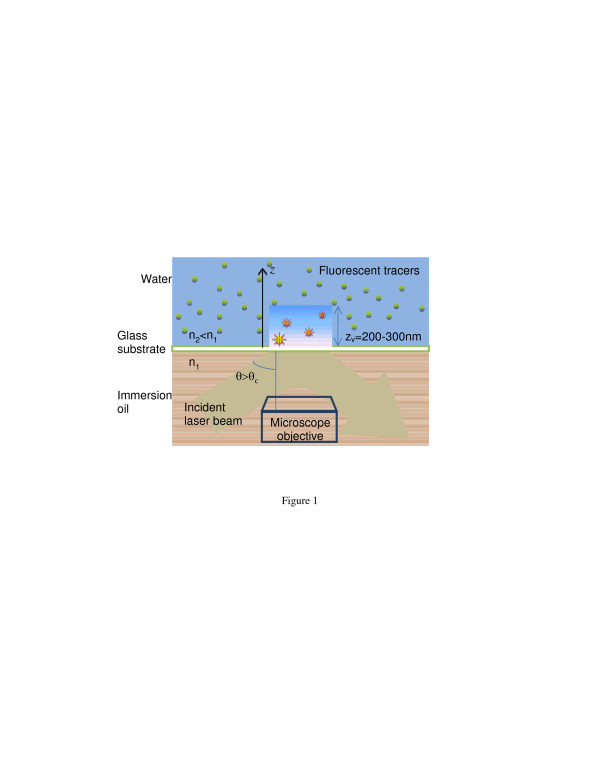
Sketch of TIR and nPIV setup.

*I*_0_ is the maximum intensity at the wall, and *z*_p_ is the penetration depth:

(2) $$z_{\text{p}}=\frac{\lambda_{0}}{4\pi n_{1}}{\left[\sin^{2}\theta -{\left(\frac{n_{2}}{n_{1}}\right)}^{2}\right]}^{-\frac{1}{2}}\text{,}$$

where *λ*_0_ is the wavelength of the light, and *θ* is the incident angle. For visible light at a glass water interface, *z*_p_ is on the order of O(100 nm) and is independent of the incident light polarization direction. It can be seen that in addition to the incident angle of the light, penetration depth depends on the refractive indices at the interface and the wavelength of light. Figure 1 shows the schematic of a TIRF setup used in a nPIV experiment where only the near-wall fluorescent particles in the fluid are excited and viewed from the bottom of the microscope plate. The emission intensity of the tracer particles in this region is also an exponential function of the distance from the wall with a decaying trend as stated by Equation 1. However, depending on the optical characteristics of the imaging system, ultimate depth of visible region, *z*_v_, depends on the intensity of the incident laser beam, fluorescent particle characteristics, camera, and the background noise of the imaging system. In practice, this depth is usually more than the estimated penetration depth.

In a μPIV experiment, the whole flow field is illuminated, and the focal depth of the microscope objective sets the out-of-plane resolution of the measurement. The emitted light from the unfocused particle tracers acts as background noise for the measurement, reducing the signal-to-noise ratio of the measurement. However, with nPIV, the focal depth of the objective lens is larger than the penetration depth of the evanescence wave; therefore, all the particles in the image are in focus, and there is no background light. The brightness (size) of the particle images is a function of their distance from the wall, where particles near the wall look bigger and brighter than those further away. The effect of this nonuniformity in the tracer brightness combined with the effect of Brownian motion and the near-wall velocity gradient is discussed in detail in a recent publication [28]. More details on TIRF, nPIV characterization, and its implementation can be found in the literature [22,24,25,29].

## Methods

### Objective-based TIRF

**Figure 2:**
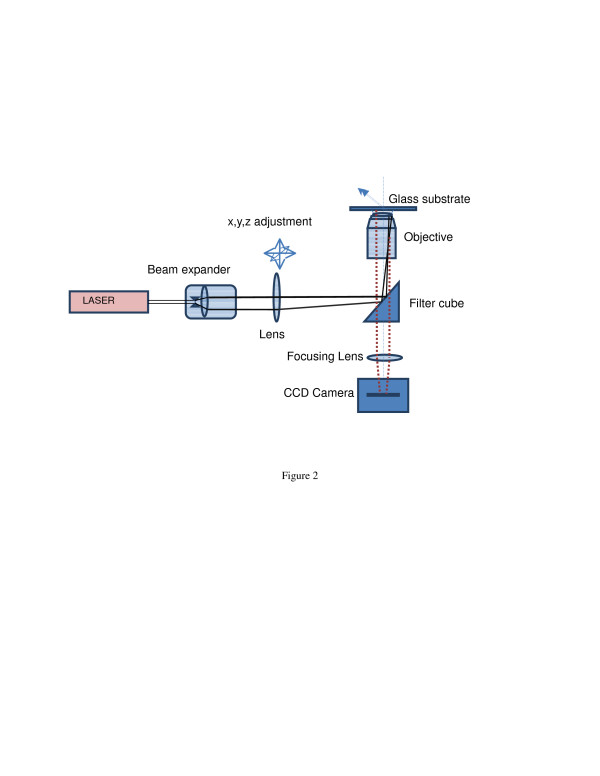
Sketch of the optical arrangement used in attaining objective-based TIRF.

Fluorescence microscopy differs from most traditional techniques in that the light of the microscope output, emitted light from the object under study, differs from the excitation light of the light source. TIRF microscopy is generally conducted in a laboratory using prism or objective-based methods. In this work, the near-wall nPIV flow measurements were carried out in a microchannel using an objective-based TIRF. Figure 2 shows the schematic of the optical arrangement used in this work. In the objective-based TIRF, the excitation laser beam first passes through a lens arrangement to fall on the dichroic filter cube placed inside the epi-fluorescence microscope. The filter cube reflects the beam at the back focal plane of the microscope objective at a point away from its axis. This puts the excitation light beam at a nonzero incident angle (*θ*) with respect to the optical axis of the objective. The emitted light from fluorescent tracer particles is then collected by the microscope objective and recorded by a camera. The dichromatic mirror removes the emission light from that of the excitation which helps in obtaining a clear image of only the tracer particles. The nanoparticles used in this work showed no fluorescent property, and the dichromatic mirror in the filter cube reflects only the excitation beam from nanoparticles and removes any possible background illumination.

### Preparation of nanofluids

One prerequisite for conducting an experimental analysis of nanofluids is to have stable and fully dispersed nanofluids. In this work, nanofluids were prepared by a top down approach in which commercially bought nanoparticles were mechanically dispersed into a base fluid (in this work, water). Ultrasonic baths and homogenizers are common tools used in breaking the agglomerates of nanoparticles compared to magnetic and high shear stirrers [30]. Stabilization of the nanofluids was then carried out using an electrostatic stabilization technique where the pH of the nanofluid suspensions was kept away from the isoelectric pH value. The pH values of the nanofluids were kept in the range of 5.5 to 6 by adding reagent grade nitric acid. In this work, initially, appropriate amounts of SiO_2_ nanoparticles (approximately 20-nm average diameter, Sigma Aldrich, #637238, St. Louis, MO, USA) were dispersed in deionized water using an ultrasonic bath (VWR ultrasonic cleaner, VWR International, LLC, Radnor, PA, USA; 35 kHz) for 30 min. Furthermore, this colloidal suspension was subjected to intensified ultrasonication by immersing a probe type sonicator (QSonica S-4000, Misonix, Qsonica, LLC, Newtown, CT, USA; 20 kHz). Cyclic ultrasonic pulses were then given to the suspension for about 30 min to achieve maximum possible de-agglomeration of particles. Four particle weight concentrations, 0.1, 0.2, 0.5 and 1 wt.%, were considered for this investigation as they exhibited acceptable optical properties required in the experiment and a good colloidal stability over time. In addition, all experiments were conducted within 1 hour from sonication time to minimize any possible chances of re-agglomeration and sedimentation.

### Experimental setup

**Figure 3:**
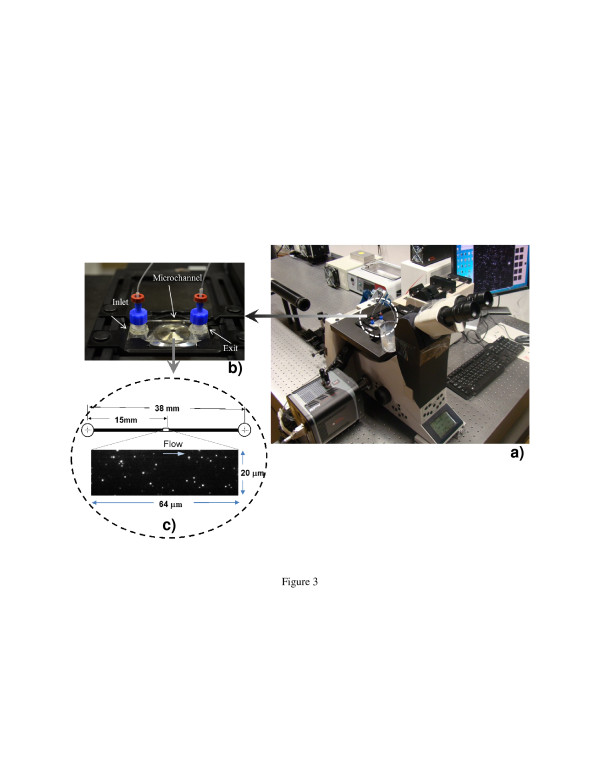
**Photographs of the experimental setup and microchannel.** (**a**) Photograph of the experimental setup; (**b**) view of microchannel with inlet and exit ports; (**c**) top view of the microchannel used in this study indicating the region of interest with a typical nPIV image obtained.

nPIV experiments are usually carried out in a quartz-liquid interface for the preferred optical property of quartz. The microchannel used in this experiment (Translume Inc., Ann Arbor, MI, USA) had a rectangular cross-section with a width and height of 300 and 100 μm, respectively, where the bottom wall of the channel is customized to have a thickness of 0.12 mm. The sidewalls of the channel deviate slightly (less than 4°) from vertical, giving the microfluidic channel a ‘nearly’ perfect rectilinear cross-section. During the experiment, different flow rates ranging from 0.02 to 0.06 ml/min (±0.1%) were maintained in the microchannel using a syringe pump (KDS200, KD Scientific, Holliston MA, USA) along with a 2.5-ml gas-tight glass syringe (Hamilton, Reno, NV, USA). The constant flow rate provided by the syringe pump passed through the microchannel via non-expanding polymer tubing and was drained into a reservoir at atmospheric pressure. Figure 3 shows the photographs of the experimental setup and the zoomed view of the microchannel placed over the microscope objective.

An argon-ion CW laser beam with a wavelength of 488 nm (Spectra Physics BeamLok 2060, Spectra Physics Inc., Santa Clara, CA, USA) was used to provide excitation light in the near-wall region. Images were obtained using an EMCCD camera (ProEM 512, Princeton Instruments, Trenton, NJ, USA) attached to an inverted epi-fluorescence microscope (Leica DMI6000B, Leica Microsystems Ltd., Milton Keynes, UK) via a 63× 1.47NA oil immersion objective. The pixel resolution for the images obtained from this imaging set up was 4 × 10^6^ (pixel/m). The nPIV seeding particles used in the flow were 100-nm-diameter (±5%) polystyrene fluorescent particles (F8803, Invitrogen, Carlsbad, CA, USA) having peak excitation and emission wavelengths of 505 and 515 nm, respectively. In all the experimental runs, the fluorescent particle concentration was maintained at a constant volume concentration of 0.017 vol.%. Fluorescent particles were added to the nanofluid suspension and sonicated when nanofluid samples were prepared. Thus, the nanofluid samples contain both SiO_2_ nanoparticles and the fluorescent particles. It should be noted that the fluorescent particles are about five times larger than nanoparticles with a concentration one order of magnitude lower than the nanoparticle concentration. This will minimize any additional particle-particle interaction between the larger seeding fluorescent and SiO_2_ particles.

Evanescent wave illumination was generated on the bottom quartz-water interface in the microchannel. The angle of incidence of light in the water-quartz interface was evaluated to be 75^o^, based on the numerical aperture value of the objective lens and refractive indices at the interface. This yielded a penetration depth of *z*_p_ ≅ 105 nm (Equation 2). The depth of visible region (*z*_v_) is then estimated to be approximately 310 ± 50 nm for the base fluid that corresponds to a non-dimensional value of *z*_v_*/h* = 3.5 × 10^−3^, where *h* is the microchannel height. This estimation is based on the penetration depth and the intensity value of the background noise in captured images. The depth of the visible region, *z*_v_, gives an estimate of the position where the velocity values are measured [25]. A typical nPIV image obtained during experimentation is shown in Figure 3c. In almost all the cases, the signal-to-noise ratio in the obtained images were more than eight and four for the base fluid and nanofluids, respectively.

For each experiment, 1,500 nPIV image pairs of 256 × 80 pixels were acquired with an inter-frame time delay of 0.6 ms. The images were then post-processed using a standard FFT-based cross correlation program that uses a 3D Gaussian peak finding algorithm based on a Gaussian surface fit to determine the tracer particles’ displacements [25]. The interrogation window size was set at 186 × 68 pixels with a search radius of 50 pixels. In each case, there were sufficient numbers of matched tracer particles in the interrogation windows. In almost all cases, the displacement was observed to be less than 5 pixels.

## Results and discussion

Experiments reported in this work were conducted in two parts: (1) nanofluids bulk flow behavior study and (2) measurement of the near-wall flow characteristics of nanofluids in a microchannel. Initially, the rheological characteristics were studied by the measurements of the nanofluid bulk viscosity. These measurements were used to investigate Newtonian characteristics of the fluid to analyze the measured near-wall velocity. It is believed that any non-homogeneous flow characteristics that may occur in nanofluids could be cross-verified by these two experiments.

### Viscosity of nanofluids

**Figure 4:**
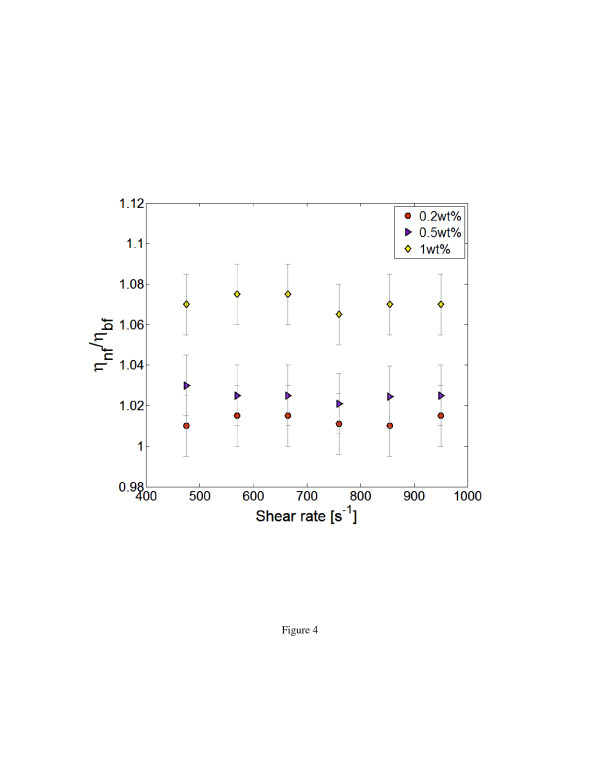
Viscosity variation for nanofluids at different shear rates (at 20°C).

The rheological characteristics of the nanofluids were measured using a Brookfield DV-111 Ultra Rheometer (Middleboro, MA, USA). The rheometer works on the basis of measuring shear stress on a rotating cone-shaped spindle immersed in the test fluid. Figure 4 shows the measured viscosity of the nanofluids (*η*_nf_) normalized by that of the base fluid (*η*_bf_) at different shear rates. The experiments were conducted at a fluid temperature of 20°C ± 0.2°C, and the error bars indicate the 95% uncertainty band of the measurement. The results of this figure are in agreement with the previous reported observations for viscosity enhancement of nanofluids for different particle loadings [31]. A closer observation of the results also shows that the nanofluids exhibited Newtonian characteristics for the range of shear rates investigated. This suggests that during near-wall fluid velocity measurement inside the microchannel, nanofluid does not show any shear thickening or thinning effects. The Newtonian behavior of SiO_2_-water nanofluids also falls in line with the recent benchmarking exercise INPBE for nanofluids [14]. Another expected feature of a Newtonian fluid is its homogeneous nature. Any systematic non-homogeneity of nanoparticle in the near-wall region that is associated with nanofluid concentration in the base fluid could affect the fluid velocity profile in this region, which is analyzed next.

### Near-wall velocity measurements for nanofluids

nPIV experiments were conducted in the microchannel for the base fluid, water, and nanofluids of different concentrations at different flow rates from 0.02 to 0.06 ml/min. Near-wall images were captured and post-processed to evaluate the averaged velocity values in the measurement region next to the wall. The measurement region was located 15 mm from the inlet port of the channel to ensure a fully developed flow condition and in the middle of the microchannel width to avoid the effects of sidewalls. All the experiments were conducted at isothermal condition with a uniform temperature of 22°C and no bubbles present in the inlet/outlet ports and flow path. After each experiment, the entire loop was cleaned thoroughly by rinsing and flushing with deionized water before the next testing fluids were introduced into the microchannel.

Since the results of the experiments for nanofluids are to be compared with that of the base fluid, efforts were taken to maintain the same experimental conditions throughout the tests. For instance, the flow loop (the syringe pump along with all the piping system), the microchannel (and its physical position on the microscope), the optical setup, and the camera settings remained constant during all the experiments. Furthermore, the optical arrangements were undisturbed, and the camera was finely focused on the bottom channel wall with the aid of the few fluorescent particles located on the wall.

**Figure 5:**
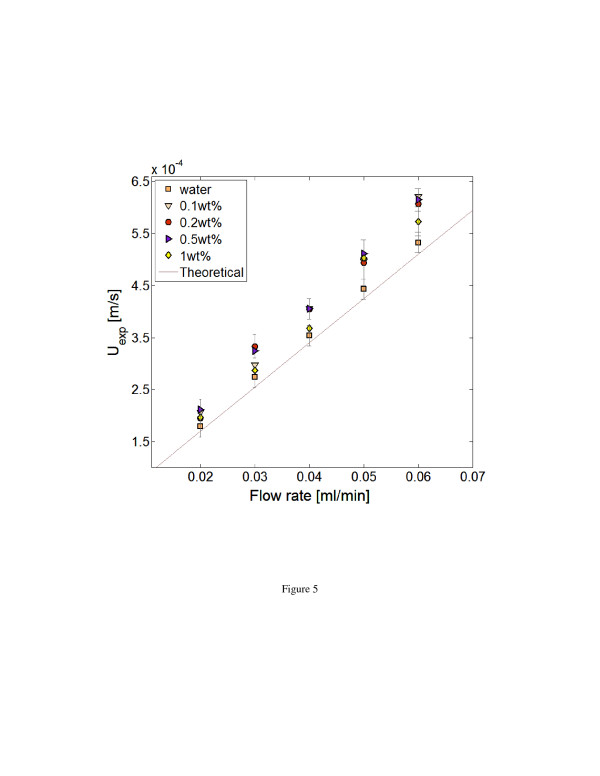
Measured near-wall velocities for water and nanofluids.

Figure 5 shows a consolidated representation of all the experimental results carried out for nanofluids, as well as the base fluid, compared to the values predicted by the analytical solution for a laminar flow. The analytical prediction is for a homogeneous fluid with Newtonian characteristics averaged in the measurement region [32]. The error bars for the selected data points represent an 85% uncertainty level that includes the measurement uncertainty sources as explained in discussion and measurement errors as suggested by Benedict and Gould [33]. As explained earlier, the out-of-plane resolution of the measurement was set by the visible region depth, *z*_v_ approximately 310 ± 50 nm. The velocity values represented in Figure 5 then depict the average velocity values at a maximum distance of 310 nm from the wall after correcting for exponential illumination intensity inherent in nPIV [28]. It is observed that the measured near-wall velocity shows a slight enhancement when compared with that of the base fluid. However, the level of enhancement for each dataset is not monotonous and does not show any clear correlation to that of nanoparticle concentration in the nanofluids. Furthermore, the velocity variations measured for all the flow rates all fall within the experimental uncertainty values of the measurement. Assuming a no-slip condition on the wall, the values of wall shear rate for the velocity data shown in Figure 5 vary from 500 to 1,500 s^−1^.

Moreover, Figure 5 shows that even after increasing the particle loading by one order of magnitude, the near-wall velocity variation is similar to that of the base fluid. This indicates that nanofluids hydro-dynamically behave as a homogeneous mixture with Newtonian fluid characteristics in the near-wall region for the length scale studied here, O(300 nm). For the current constant flow rate setup, the increase in the viscosity of the fluid results only in an increase in pressure drop across the channel and does not affect the nanofluid velocity profile. However, in the case of aggregation of particles in nanofluids, the measured near-wall velocities would be different when compared with that of the theoretical and experimental base fluid as a result of non-homogeneous nature of the flow. Micro-PIV results of Walsh et al. [20] point to the same conclusion for nanofluid flow inside a microchannel. However, the present nPIV results provide insight in the region very close to the wall. Even though nPIV technique enables us to obtain velocities within O(100 nm) of the wall, more analysis and attention needs to be given to various factors affecting the measurement methodology, which is briefly described next.

### Experimental uncertainties

Before concluding remarks, it is necessary to discuss possible limitations of the nPIV method for the measurement of the near-wall velocities for nanofluids. One major source of error that may occur is due to the change in refractive indices of the nanofluids at various concentrations. The measured velocity represents some average velocity of the tracer particles in the observation region, *z*_v_, as defined by the out-of-plane resolution of the measurement, *z*_p_. Since the penetration depth of the evanescent wave is a function of the refractive index of the fluid (Equation 2), any misjudgments in the optical properties of the nanofluids can introduce a bias in the analysis. A recent experimental study by Kim and Kihm [34] investigating the effective refractive index of nanofluids using a total internal reflectometer revealed a negligible increase in the refractive index values with an increase in nanoparticle concentration. They show that for Al_2_O_3_-water nanofluids, the refractive index increased from 1.332 to 1.335 as the particle concentration increased from 0 to 1 vol.%. Their experimental observation matched well with the refractive index predictions obtained from the Rayleigh scattering theory for colloids. A similar theoretical observation can be seen for various nanofluids in an optical property characterization study of Taylor et al. [35]. Therefore, it can be concluded that for the present work, which has a maximum particle volume concentration of 0.25 vol.%, the bias that may occur due to the variation in the refractive index of the sample is negligible.

With the addition of nanoparticles to the base fluid, the optical transparency of the nanofluids suspension is decreased. Consequently, the practical depth of visibility, *z*_v_, would be reduced with the increase in the nanofluid concentration. For the shear flow near the wall, when *z*_v_ is reduced, only particles closer to the wall are visible and participate in the velocity measurement. Since the fluid velocity is slower in the region closer to the wall, the measured averaged velocity is expected to be smaller than the average velocity obtained for the case of the larger, *z*_v_. The observed reduction of the measured velocities for the highest particle concentration of 1 wt.% in Figure 5 may be an indication of this phenomenon. To avoid this complication, only particle concentrations of 1 wt.% and less were considered in this work, and the obtained data all fall into the uncertainty range of the measurement. In the future, experimental investigations will be required to evaluate the effect of particle type and concentration on the visible depth and the subsequent nPIV velocity measurement.

Another factor that can affect the quality of PIV images (signal-to-noise ratio) is the effect of SiO_2_ nanoparticle depositing on the bottom wall of the channel where the measurement is made. Sedimentation on the bottom channel wall can introduce more noise thereby reducing the image quality and signal-to-noise ratio. As the nanofluids used were thoroughly sonicated just before the experiments, this effect was highly reduced. In addition, care is taken such that velocity experiments were conducted in a shorter period of time, and the flow was not stopped in between for longer durations, which could enhance particle deposition at the bottom walls.

Distinct from the above stated biases, the nPIV experimental observations at the near-wall region suggest that nanofluids behave as a homogeneous mixture and have Newtonian characteristics. This indirectly suggests that any convection heat transfer enhancement associated with nanofluids may be caused due to the augmentation of nanofluid properties following effective medium theory. However, the above statement should be taken with some skepticism as the present experiments were done at constant temperatures, and no heat transfer effects occurred near the wall. Experimental efforts are presently underway in our laboratory to improve measurement uncertainties and further investigate the effect of heat transfer in this region.

## Conclusions

The present study outlines for the first time implementation of the state-of-the-art nPIV technique for investigating the near-wall flow characteristics of nanofluids. SiO_2_-water nanofluids were used in the study with particle weight concentrations varying from 0.1 to 1 wt.%. Near-wall velocities of nanofluids were then measured for a range of flow rates, and the results were then compared with that of analytical and experimental values for the base fluid (water). It is observed that even though nanofluid viscosities increased with particle loading, the near-wall velocity values were similar to that of the base fluid in the range of the experimental uncertainties. This observation could be attributed to the homogenous nature and Newtonian characteristics of the suspension. Even though only cold flow studies were made in the present work, it is believed that utilizing this near-wall measurement technique might yield more insight into the flow physics of nanofluids, facilitating improvements in a proposed theoretical model based on experimental observations.

## Abbreviations

μPIV, microparticle image velocimetry; SiO2, silicon dioxide; TIR, total internal reflection; TIRF, total internal reflection fluorescence.

## Competing interests

The authors declare that they have no competing interests.

## Authors’ contributions

AK conducted the experiments and drafted the manuscript. RS supervised the whole work starting from design to analysis of data and edited the manuscript. Both authors read and approved the final manuscript.

## Authors’ information

AK is a post-doctoral research associate at the Mechanical Engineering Program, Texas A&M University at Qatar, Doha, Qatar. RS is an assistant professor at the Mechanical Engineering Program, Texas A&M University at Qatar, Doha, Qatar
